# The Role and Feasibility of Automated Breast Ultrasound in the Evaluation of Male Breast Disease: Workflow Efficiency and Imaging Spectrum

**DOI:** 10.7759/cureus.103467

**Published:** 2026-02-12

**Authors:** Abdulkadir Eren, Emrah Karatay, Irmak Durur-Subasi

**Affiliations:** 1 Radiology, Istanbul Medipol University Mega Hospital, Istanbul, TUR; 2 Radiology, Sultan II. Abdulhamid Han Training and Research Hospital, Istanbul, TUR; 3 Radiology, Acibadem University, Istanbul, TUR

**Keywords:** anatomical plane (c-plane), automated breast ultrasound, bi-rads, gynecomastia, male breast cancer, triage workflow efficiency

## Abstract

Traditional hand-held ultrasonography (HHUS) suffers from inherent limitations, including operator dependency, lack of reproducibility, and long acquisition times in busy clinical settings. Automated breast ultrasound (ABUS) addresses these drawbacks by providing standardized, three-dimensional, and operator-independent imaging that separates acquisition from interpretation. While the diagnostic performance of ABUS has been extensively validated for supplemental screening in females with dense breast tissue, its specific application in the male population remains an unexplored frontier in the literature.

Objective

The objective of this study was to evaluate the diagnostic value, workflow advantages, and imaging spectrum of ABUS in male breast diseases based on a large-scale, single-center experience.

Methods

This retrospective study included 85 male patients (mean age: 36.6 ± 13.7 years) who underwent ABUS between April 2023 and February 2025. Inclusion criteria focused on symptomatic patients and those requiring high-risk screening or follow-up. Recall rates, complementary imaging frequency, and the Breast Imaging Reporting and Data System or BI-RADS categories were analyzed.

Results

Breast pain (53 cases, 62.4%) was the most common symptom, and gynecomastia (29 cases, 34.1%) was the most frequent finding. The recall rate for secondary HHUS was 1.2% (1 case). Three biopsies revealed one lipoma, one invasive ductal carcinoma, and one papillary carcinoma.

Conclusion

ABUS provides a standardized, reproducible evaluation of male breast diseases with minimal recall and optimized workflow efficiency. It serves as a promising adjunct to standard imaging protocols, particularly in younger males where avoiding radiation is preferable, and offers a unique anatomical plane (C-plane).

## Introduction

Male breast disease, while significantly less common than in females, poses a distinct clinical challenge due to the rarity of malignancies and the predominance of benign conditions such as gynecomastia. Although male breast cancer (MBC) accounts for less than 1% of all breast cancer cases globally, its incidence has been slowly rising over the last decades [1]. Unlike female breast cancer, which benefits from established screening programs, male breast malignancies often present at more advanced stages - frequently with nipple involvement or chest wall invasion - due to a lack of routine screening, delayed presentation, and lower clinical awareness among both patients and healthcare providers.

The diagnostic evaluation of the male breast has traditionally relied on a combination of clinical examination, mammography, and hand-held ultrasonography (HHUS). According to the American College of Radiology (ACR) Appropriateness Criteria (2018/2024), mammography remains the standard and primary imaging tool for symptomatic men aged 25 years and older [2]. Mammography is excellent for characterizing calcifications; however, in the male breast, the paucity of tissue can make positioning difficult and painful, often leading to suboptimal imaging of the posterior axillary line. Furthermore, for younger men or those with painful gynecomastia, the compression required for mammography is a significant deterrent. In contrast, ultrasonography (US) is recommended as the initial modality for younger patients or as a supplementary tool to characterize indeterminate mammographic findings and guide biopsies [3].

US is established as a cornerstone for evaluating the male breast because it is non-invasive, cost-effective, and lacks ionizing radiation. It is particularly effective in distinguishing between the retroareolar, flame-shaped density of true gynecomastia and the eccentric, irregular hypoechoic masses typical of carcinoma. Despite its utility, traditional HHUS suffers from inherent limitations, including operator dependency, lack of reproducibility, and long acquisition times in busy clinical settings [4]. These factors can lead to variability in lesion characterization and potential missed diagnoses, especially in centers with varying levels of radiologic expertise. Automated breast ultrasound (ABUS) addresses these drawbacks by providing standardized, three-dimensional (3D), and operator-independent imaging that separates acquisition from interpretation [5]. Originally developed to screen women with dense breast tissue (ACR density C and D), ABUS uses a large-format (typically 15-17 cm) linear transducer to capture high-resolution volumetric data. This technology offers a unique advantage: the coronal plane [6]. Often dubbed the "anatomical plane" or "C-plane," the coronal view is reconstructed from the 3D dataset and allows the radiologist to view the breast tissue parallel to the chest wall. This view is superior for visualizing architectural distortions and the "retraction phenomenon" often observed in malignancies, which can be subtle or invisible on standard 2D HHUS [7]. While the diagnostic performance of ABUS has been extensively validated for supplemental screening in women with dense breast tissue, its specific application in the male population remains an unexplored frontier in the literature [8].

The anatomical simplicity of the male breast - lacking the complex lobular overlapping found in females - suggests that men may derive significant benefit from the wide field-of-view offered by ABUS. Furthermore, the shift from a radiologist-performed HHUS to a technician-performed ABUS workflow offers profound operational benefits, potentially reducing wait times and allowing for "appointment-free" imaging models. The limited breast volume in men suggests they may derive significant benefit from the wide field-of-view and multiplanar reconstructions (MPRs) offered by ABUS, which enhance the detection of distortions and subtle lesions that might be overlooked during a traditional sweep of a hand-held probe [9]. Our institution integrated ABUS into routine male breast assessment immediately following its installation to streamline clinical operations and standardize diagnostic quality. This study retrospectively evaluates our large-scale, single-center experience - representing one of the most comprehensive cohorts to date - focusing on the diagnostic spectrum of findings, recall rates, and the potential of ABUS to serve as a primary triage tool for younger male patients. By re-evaluating this traditional diagnostic algorithm, we aim to demonstrate the tangible clinical workflow advantages and expanded clinical application of ABUS beyond female screening.

## Materials and methods

Study population and ethics

This retrospective, single-center study was approved by the Istanbul Medipol University Ethics Committee (IRB no: E-10840098-202.3.02-7201). The study adhered to the principles of the Declaration of Helsinki. We performed a comprehensive database search to identify all male patients who underwent breast imaging between April 2023 and February 2025. From this initial pool, we identified 85 consecutive male patients who underwent ABUS as their primary or supplemental imaging modality.

Inclusion and exclusion criteria

The following have been defined as inclusion criteria: male patients aged 14 years or older, patients presenting with clinical symptoms such as palpable mass, breast enlargement (gynecomastia), mastodynia (breast pain), nipple discharge, symptomatic patients referred for high-risk screening (e.g., *BRCA2* mutation carriers or strong family history), or follow-up of previously known benign lesions.

Three main criteria have been identified as exclusion criteria: patients with a history of mastectomy or extensive chest wall surgery that prevented proper transducer coupling, incomplete clinical or radiological records, and technical failure (e.g., motion artifacts) preventing diagnostic Breast Imaging Reporting and Data System (BI-RADS) assessment.

Imaging technique and acquisition protocol

Examinations were performed using the Invenia™ ABUS 2.0 system (GE Healthcare, Milwaukee, WI, USA). This system uses a wide-bandwidth (6-15 MHz) linear array transducer specifically designed to conform to the breast's curvature. Patients were positioned supine with the ipsilateral arm raised above the head and the hand resting behind the neck. This position flattens the breast tissue against the chest wall, minimizing thickness and improving sound wave penetration to the pectoral muscle. A standard lotion was applied for acoustic coupling. The automated transducer applied distinct compression (typically 2-4 lbs) to ensure image uniformity. Three standard views were obtained for each breast when tissue volume permitted: antero-posterior (AP), lateral (LAT), and medial (MED). To prevent nipple shadowing and the retroareolar "blind spot" often encountered in male imaging, a generous amount of ultrasound gel was applied, avoiding air gaps between the probe and the skin. The device automatically adjusted the pressure to ensure image optimization and patient comfort.

**Figure 1:**
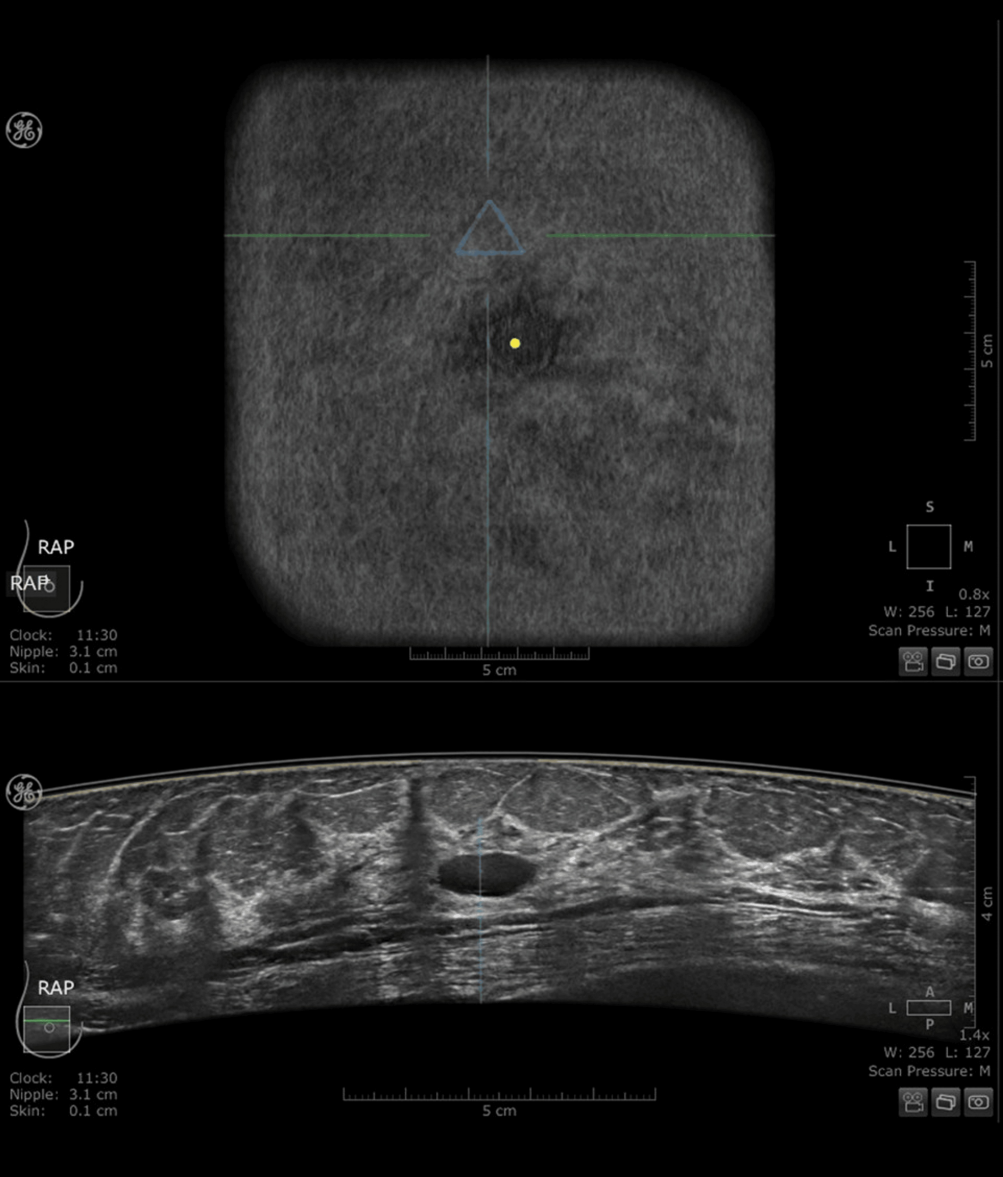
Representative ABUS workstation view demonstrating the digital tagging protocol for symptomatic patients. The top image (coronal plane) displays the dedicated "triangle" icon placed by the technologist to mark a palpable mass at the 11:30 clock position. The bottom image (transverse view) reveals the corresponding lesion directly aligned with the marker, facilitating correlation between the physical examination finding and the 3D volume review. 3D, three-dimensional; ABUS, automated breast ultrasound.

However, due to the smaller volume of the male breast, the protocol was tailored; a single centralized AP view often sufficed for complete coverage. The median number of views was 4 (interquartile range: 3) per patient (total for both breasts). All scans were performed by certified radiology technicians who had completed specific training on ABUS positioning. Before the scanning procedure, trained technicians obtained a focused anamnesis and performed a physical palpation to identify areas of pain, hardness, or palpable masses. Any symptomatic area identified during this preliminary examination was digitally marked on the acquisition screen using the system’s dedicated "triangle" icon. These digital markers were visible to the radiologist during the workstation review, facilitating a direct correlation between the physical findings and the 3D volume, effectively bridging the gap created by the absence of a real-time physician-performed examination (Figure 1).

Image analysis and interpretation

The 3D volumetric datasets were sent to a dedicated workstation (Invenia ABUS Workstation). Images were reviewed by two radiologists with 10 and 15 years of experience in breast imaging, respectively. The interpretation process used the MPR capabilities, allowing simultaneous review of axial, sagittal, and coronal planes. Lesions were characterized according to the BI-RADS 5th edition lexicon: (1) gynecomastia was classified into nodular, dendritic, or diffuse glandular patterns; (2) masses were evaluated for shape, orientation, margin, echo pattern, and posterior acoustic features; and (3) recall was defined as any case where the ABUS findings were indeterminate (BI-RADS 0), requiring the patient to return for additional HHUS or mammography for clarification.

Statistical analysis

Descriptive statistics were used to summarize the demographic and clinical data. Continuous variables (e.g., age) were expressed as mean ± standard deviation. Categorical variables (e.g., BI-RADS category and symptoms) were presented as frequencies and percentages (%). All statistical analyses were performed using SPSS version 26.0 (IBM Corp., Armonk, NY).

## Results

A total of 85 male patients were included in the final analysis. The mean age of the cohort was 36.6 ± 13.7 years, with a range of 14 to 78 years. The age distribution showed a bimodal tendency, with peaks in late adolescence (pubertal gynecomastia) and middle age.

Diagnostic concordance and BI-RADS distribution

A significant proportion of findings were categorized as BI-RADS 3 (60.0%). This elevated rate of "probably benign" categorization, rather than BI-RADS 2, reflects the inherent limitation of ABUS in lacking real-time Doppler or elastography features. Without these tools to immediately confirm avascularity or soft tissue elasticity, benign-appearing solid masses were cautiously categorized as BI-RADS 3 to ensure short-term stability before final discharge.

Symptomatology

Breast pain (mastodynia) was the predominant presenting symptom, reported by 53 patients (62.4%). This high prevalence of pain correlates strongly with the acute phase of gynecomastia development. A palpable mass was reported by 16 (18.8%) patients, who presented specifically complaining of a "lump," breast enlargement was reported by 18 (21.2%) patients, who reported generalized enlargement, and 15 (17.6%) patients were asymptomatic, who were referred largely for follow-up of prior findings or incidental notes from other chest imaging (e.g., CT).

Physical examination revealed a palpable mass in 27 patients (31.8%), which is higher than the patient-reported lump rate, suggesting that many patients with gynecomastia perceive it as generalized enlargement rather than a distinct mass (Table 1).

**Table 1:** Patient characteristics and clinical presentation

Variable	Value (n=85)
Age (years, mean ± SD)	36.6 ± 13.7
Presenting symptom	
Breast pain	53 (62.4%)
Breast enlargement	18 (21.2%)
Palpable mass	16 (18.8%)
Asymptomatic	15 (17.6%)
Physical examination findings	
Palpable mass	27 (31.8%)
Asymmetry/nipple retraction	11 (12.9%)
Palpable axillary mass	8 (9.4%)

**Figure 2:**
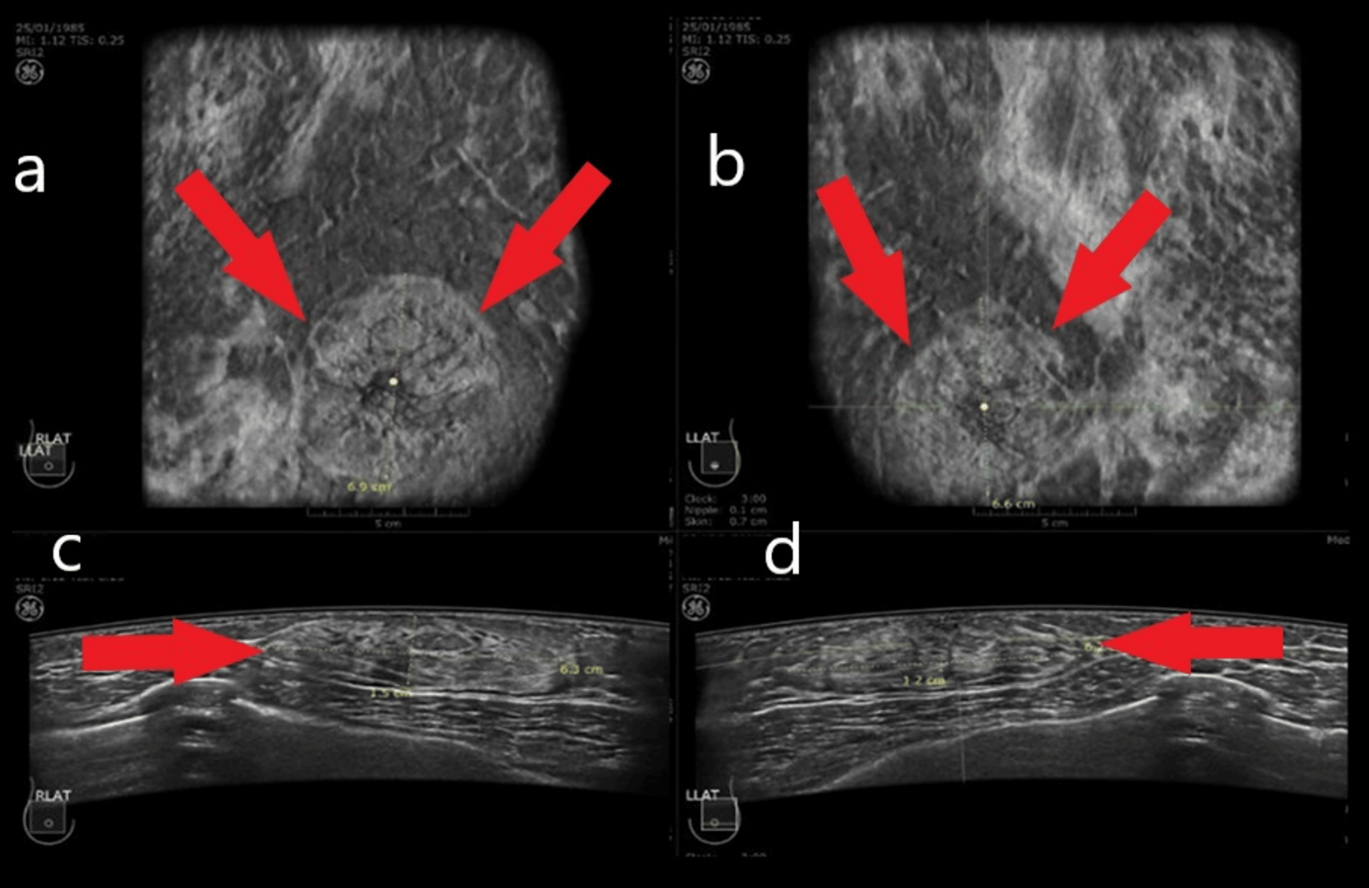
Bilateral nodular gynecomastia (red arrow) is observed on coronal (a and b) and transverse images (c and d). Due to wide coverage, all the fibroglandular tissues have been covered.

The distribution of findings highlights the benign nature of male breast disease in this cohort. (1) Gynecomastia (34.1%, n=29 cases): This was the most frequent finding. ABUS excelled in differentiating the subtypes. The dendritic type (flame-shaped) was the most common, appearing as a retroareolar hypoechoic area with finger-like projections extending into the surrounding fat. The nodular type appeared as a more circumscribed, spherical, or subareolar opacity (Figure 2). The coronal plane was particularly useful here. It visualized the "spider-leg" appearance of dendritic gynecomastia in a single view, confirming its benignity without the need for Doppler interrogation. (2) Normal/negative (37.6%, n=32 cases): A significant portion of patients presenting with pain had BI-RADS 1 (negative) or BI-RADS 2 (benign, e.g., simple cysts and intramammary lymph nodes) findings. In these cases, ABUS provided confident exclusion of pathology, reassuring the patient without further intervention. (3) Masses (18.8%, n=16 cases): Solid masses identified included lipomas, sebaceous cysts, and the malignant cases detailed below. ABUS findings and BI-RADS distributions are presented in Table 2.

**Table 2:** ABUS imaging findings and BI-RADS categories ABUS, automated breast ultrasound; BI-RADS, Breast Imaging Reporting and Data System; HHUS, hand-held ultrasonography.

Variable	Value (n=85)
ABUS finding	
Gynecomastia (dendritic/nodular)	29 (34.1%)
Mass	16 (18.8%)
Normal/negative	32 (37.6%)
BI-RADS category	
BI-RADS 2	32 (37.6%)
BI-RADS 3	51 (60.0%)
BI-RADS 5	1 (1.2%)
BI-RADS 6	1 (1.2%)
Recall and secondary imaging	
Recall for HHUS	1 (1.2%)
Complementary mammography	2 (2.4%)
Complementary MRI	2 (2.4%)

Regarding histopathology, in all three cases where biopsy was performed (one lipoma, one invasive ductal carcinoma (IDC), and one papillary carcinoma), the ABUS BI-RADS assessment was pathologically concordant. The system correctly identified suspicious morphological features in the malignant cases, triggering appropriate biopsies. Additionally, seven patients underwent surgery for malignant and symptomatic benign conditions.

**Figure 3:**
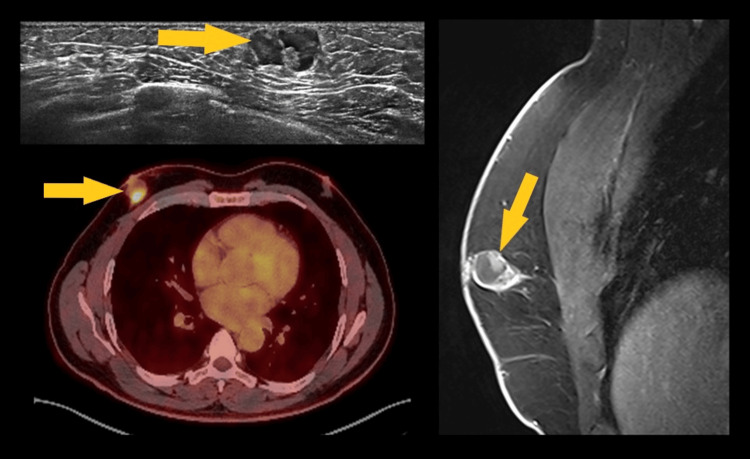
Transverse ABUS image (left top) shows the heterogeneous, well-bordered mass (papillary carcinoma). The mass uptakes the radiotracer on positron emission tomography (left bottom) and is observed as a complex cyst on MRI (right). The described lesion is highlighted with a yellow arrow.

Pathological verification (core needle biopsy or excision) was performed in three cases: (1) IDC: One patient (68 years old) presented with a painless, hard retroareolar mass. ABUS demonstrated an irregular, vertically oriented hypoechoic mass with posterior acoustic shadowing. Crucially, the coronal view showed the "retraction phenomenon" - a pulling in of the surrounding Cooper's ligaments - which is a hallmark of malignancy. This was classified as BI-RADS 5. (2) Papillary carcinoma: A 55-year-old male presented with bloody nipple discharge. ABUS revealed a complex cystic and solid mass within a dilated duct, classified as BI-RADS 4C (Figure 3). (3) Lipoma: One soft, palpable mass was confirmed as a lipoma. On ABUS, this appeared as an isoechoic, encapsulated mass parallel to the skin, easily distinguished from glandular tissue.

The most striking result of this study is the low recall rate. Only 1 (1.2%) patient required a recall for secondary HHUS to clarify a finding observed on ABUS. Complementary mammography was requested for only 2 (2.4%) patients, primarily to investigate microcalcifications in the malignant cases. MRI was used in two cases for staging.

By using technicians for the scanning process, radiologist involvement was limited to interpretation. The average interpretation time for a male ABUS study was less than three minutes, significantly faster than the 10-15 minutes typically reserved for a radiologist-performed HHUS. These findings suggest that ABUS is a highly efficient tool for distinguishing between gynecomastia and suspicious masses, effectively optimizing clinical workflow.

## Discussion

The findings of this study validate ABUS as a highly effective, robust, and workflow-efficient modality for the evaluation of male breast disease. While ABUS is FDA-approved for screening women with dense breasts, our data suggest a compelling expanded clinical application in the male population, characterized by a recall rate (1.2%, n=1 case) that is drastically lower than those reported in female screening trials (often 5%-15%) [10]. While the diagnostic performance of ABUS has been extensively documented in female populations - primarily as a supplemental tool for dense breasts - this study represents one of the largest single-center experiences systematically applying ABUS to a male cohort.

The male breast anatomy is distinct; it is predominantly fatty with a rudimentary ductal system and lacks the physiological changes of lobular development observed in women. This "anatomical simplicity" is a double-edged sword for HHUS but an advantage for ABUS. In HHUS, the operator must apply pressure to maintain contact, which can compress the retroareolar tissue, potentially obscuring the vascularity or shape of small gynecomastia discs. ABUS, with its large footprint and standardized compression, captures the breast in a neutral state. Our study highlights the specific value of the anatomical plane (C-plane). In gynecomastia, the distinction between "nodular" (mass-like) and "dendritic" (infiltrative) patterns is crucial.

Dendritic gynecomastia can mimic the spiculation of cancer on axial two-dimensional (2D) images. However, on the ABUS coronal view, gynecomastia typically displays a symmetrical, "fan-shaped" distribution radiating from the nipple, whereas malignancies (like the IDC case in our cohort) present as an eccentric, chaotic distortion with converging lines. This visualization likely contributed to our high specificity and low recall rate [11].

Perhaps the most significant implication of our study is operational. Traditional male breast imaging is resource-intensive. A radiologist-performed HHUS requires the physician to be physically present for 15-20 minutes per patient. In a high-volume center, this creates bottlenecks. By implementing ABUS, we decoupled acquisition from interpretation.

Appointment-free access

Because technicians perform the scan, we could offer walk-in services for men with breast pain, significantly increasing patient satisfaction.

Radiologist time

The radiologist reviews the volumetric dataset at a dedicated workstation. For a male breast, which has less volume than a female breast, this review takes 2-3 minutes. This represents a 400%-500% increase in radiologist time efficiency compared with HHUS [12].

Standardization

One of the main criticisms of HHUS is that "if you did not save the image, it did not happen." ABUS saves the entire volume. This allows for retrospective review and precise comparison if the patient returns with increased pain or enlargement six months later, eliminating the "subjectivity gap" of different operators [5,13].

While the transition to a technician-driven model raises valid concerns regarding the loss of real-time radiologist palpation, our protocol mitigates this through mandatory technician training in physical examination and the use of digital skin markers. This ensures that the "area of concern" is highlighted on the workstation, maintaining clinical-radiological correlation.

Current ACR guidelines recommend mammography for symptomatic men >25 years old. The rationale is that mammography is more sensitive for calcifications (ductal carcinoma in situ). However, MBC is overwhelmingly IDC, which almost always presents as a mass detectable by ultrasound. Our study challenges the "mammography-first" dogma for young and middle-aged men (mean age: 36.6 years). Mammography in men is often painful due to the need to pull the pectoralis muscle into the bucky to image the small amount of breast tissue.

Radiation sparing

In our cohort, 34% of patients had gynecomastia. Exposing these men, many of whom are under 40, to ionizing radiation via mammography provides little diagnostic gain over ultrasound. ABUS offers a radiation-free alternative that is more comfortable and likely equally sensitive for the mass-forming lesions typical of male pathology.

Triage tool

We propose that ABUS should be the primary triage tool for men under 40. Mammography should be added only if ABUS demonstrates suspicious microcalcifications or indeterminate architectural distortion. Our low mammography utilization rate (2.4%) supports this tiered approach.

Historically, studies evaluating male breast imaging have focused on the diagnostic accuracy of mammography and HHUS [14]. For instance, Adıbelli et al. emphasized that the combination of mammography and US provides the highest diagnostic yield in men [18]. However, traditional HHUS is limited by operator dependency and a lack of standardized archiving. What distinguishes our study is the transition from radiologist-led HHUS to technician-led ABUS. By adopting this model, we optimized clinic throughput, allowing male patients to be examined without prior appointment scheduling due to the brevity and standardization of acquisition. This "appointment-free" integration is a significant workflow advantage not previously detailed in male breast imaging literature.

The spectrum of findings in our cohort aligns with established literature on male breast pathology, where benign conditions predominate. Gynecomastia was the most frequent diagnosis (34.1%), predominantly of the dendritic type. This spectrum aligns with established reports, confirming that benign processes represent the vast majority of male breast pathologies [15]. These results are consistent with prior reports by Nguyen et al. and Gunhan-Bilgen et al., which identify gynecomastia as the leading cause of male breast symptoms [3,15]. ABUS MPRs provided precise retroareolar evaluation, which is crucial for distinguishing nodular gynecomastia from early-stage malignancy [3,16]. While we identified two rare malignancies, the low incidence is consistent with global epidemiology, where MBC accounts for less than 1% of all breast cancers [17]. One of the most significant observations in our study relates to clinical workflow efficiency. Traditional HHUS is inherently operator-dependent and time-consuming, requiring the presence of a radiologist for both acquisition and interpretation. By integrating ABUS, we were able to separate these two phases. Trained technicians performed the standardized scans, allowing the institution to offer "appointment-free" imaging for male patients. This optimized radiologist throughput and significantly reduced patient waiting times, a practical benefit that is crucial in high-volume clinical settings.

We acknowledge that according to the 2018 ACR guidelines, mammography remains the first-line choice for males over 25 [18]. In our cohort, which had a mean age of 37 years, ABUS was used as the primary tool due to institutional protocol. Despite this divergence from traditional "mammography-first" pathways, the minimal requirement for complementary mammography (2.4%, n=2 cases) and MRI (2.4%, n=2 cases) in our results suggests that ABUS is a highly reliable triage tool. In younger populations or centers with limited access to mammography, ABUS may serve as a potent primary assessment tool with high diagnostic yield.

Limitations

This study has several limitations. The retrospective, single-center design may introduce selection bias. The relatively young mean age of our cohort (37 years) likely contributed to the low recall rate and high prevalence of benign findings, which may not be fully generalizable to older, higher-risk populations. We acknowledge that in older populations with higher cancer prevalence, the recall rate and need for complementary imaging might be higher. Additionally, the small number of malignant cases (n=2) restricts our ability to perform robust statistical analysis of diagnostic accuracy. Furthermore, the lack of real-time elastography and Doppler imaging on the ABUS platform contributed to a higher rate of BI-RADS 3 classifications compared with standard HHUS, necessitating short-term follow-up for findings that might otherwise be dismissed as benign. Finally, while technicians were trained to mark palpable abnormalities, the absence of a radiologist-performed physical examination remains a divergence from the traditional gold standard, potentially affecting the immediate characterization of subtle palpable findings.

## Conclusions

ABUS is a feasible, efficient, and diagnostically robust tool for evaluating the male breast. It combines the resolution of ultrasound with the standardization of CT/MRI. By facilitating a technician-driven workflow and offering the unique diagnostic perspective of the coronal plane, ABUS has the potential to replace HHUS as the first-line modality for symptomatic males, particularly in centers managing high patient volumes.
